# PfHMGB2 has a role in malaria parasite mosquito infection

**DOI:** 10.3389/fcimb.2022.1003214

**Published:** 2022-11-25

**Authors:** Sudhir Kumar, Stefan H. I. Kappe

**Affiliations:** ^1^ Center for Global Infectious Disease Research, Seattle Children’s Research Institute, Seattle, WA, United States; ^2^ Department of Pediatrics , University of Washington, Seattle, WA, United States; ^3^ Department of Global Health, University of Washington, Seattle, WA, United States

**Keywords:** gametocyte, differentiation, transmission, mosquito, oocyst

## Abstract

Differentiation of asexually replicating parasites into gametocytes is critical for successful completion of the sexual phase of the malaria parasite life cycle. Gametes generated from gametocytes fuse to form a zygote which differentiates into ookinetes and oocysts. The sporozoites are formed inside oocysts which migrate to the salivary glands for next cycle of human infection. These morphologically and functionally distinct stages require stage-specific gene expression *via* specific transcriptional regulators. The capacity of high mobility group box (HMGB) proteins to interact with DNA in a sequence independent manner enables them to regulate higher order chromosome organization and regulation of gene expression. *Plasmodium falciparum* HMGB2 (*Pf*HMGB2) shows a typical L- shaped predicted structure which is similar to mammalian HMG box proteins and shows very high protein sequence similarity to *Py*HMGB2 and *Pb*HMGB2. Functional characterization of *Pf*HMGB2 by gene deletion (*Pfhmgb2¯*) showed that knockout parasites develop normally as asexual stages and undergo gametocytogenesis. Transmission experiments revealed that *Pfhmgb2¯* can infect mosquitoes and develop as oocyst stages. However, transmission was reduced compared to wild type (WT) parasites and as a consequence, the salivary gland sporozoites were reduced in number. In summary, we demonstrate that *PfHMGB2* has no role in asexual growth and a modest role in sexual phase development and parasite transmission to the mosquito.

## Introduction

*Plasmodium falciparum* (*Pf*) is a digenetic parasite which completes its life cycle in the human host and a female *Anopheles* mosquito. The development inside humans involves exoerythrocytic forms which transitions from asexual reproduction in hepatocytes to asexual schizogony in erythrocytes. During erythrocytic stage of infection, a small fraction of asexually replicating parasites commit to sexual development and differentiate into sexual stages called gametocytes. Inside the mosquito midgut, the gametocytes taken up during an infectious blood meal get rapidly activated to form gametes, fuse to form short-lived zygotes which differentiate into motile ookinetes and penetrate the midgut epithelium wall to develop as oocysts. Oocyst form sporozoites which take residence in the salivary glands and are eventually injected into a new human host by bite.

The development and differentiation of *P. falciparum* sexual stages involves expression of specific transcripts (López-Barragán et al., 2011; van Biljon et al., 2019). The ApiAP2 family members have been studied for their role in transcriptional regulation (Kafsack et al., 2014; Shang et al., 2021; Singh et al., 2021). *Pf*AP2-G is required for initial commitment of the asexual parasites into gametocytes and regulates additional transcriptional regulators (Llorà-Batlle et al., 2020). Sexual development also involves sex-specific transcript expression (Le Roch et al., 2004; Khan et al., 2005; Young et al., 2005) and translational repression of mRNAs *via* an RNA helicase DOZI (development of zygote inhibited) (Mair et al., 2006). Recent studies have also indicated the role of putative DNA and RNA binding proteins in regulating gamete fertility of *P. falciparum* and the rodent malaria *P. berghei* parasites (Russell et al., 2021; Kumar et al., 2022a; Kumar et al., 2022b).

Proteins belonging to the HMGB family (high mobility group box) (PFAM ID PF00505) are eukaryotic non-histone nuclear proteins which maintain the structure and function of chromosomes. HMGB proteins have multiple functions including DNA replication, transcription, and recombination (Travers, 2003). Mammalian HMGB1 is also secreted as a damage-associated molecular pattern (DAMP) molecule regulating inflammation and immune responses (Chen et al., 2022). Mammalian HMGB2 was initially identified as a male fertility regulator with high expression in lymphoid organs and testes (Ronfani et al., 2001). HMGB2 also plays a key role in spermatogenesis in turtles (Li et al., 2022). *P. falciparum* has four HMGB family proteins (*Pf*HMGB1 - 4), which show transcriptional expression during the erythrocytic stages (López-Barragán et al., 2011). *Pf*HMGB1 and *Pf*HMGB2 proteins are expressed by both asexual and sexual stages, possess DNA binding affinities (Briquet et al., 2006), and are also secreted and are potent inducers of pro-inflammatory cytokines (Kumar et al., 2008). *Pf*HMGB1 regulates gene expression *via* mediating the structural organization of the genome but is dispensable for asexual growth of parasites (Lu et al., 2021). Studies in rodent malaria parasite *P. yoelii* have demonstrated a role for HMGB2 in mosquito infection (Gissot et al., 2008) while in *P. berghei* it acts as an alarmin contributing to the cerebral malaria (Briquet et al., 2015) and confers long-lasting protection in a murine experimental cerebral malaria (Briquet et al., 2020).

*Pf*HMGB2 shows higher expression in sexual stages (López-Barragán et al., 2011). It is reported to be refractory to gene deletion (Lu et al., 2021), although a piggy bac mutagenesis study was able to report disruption and parasite survival with a compromised growth (Zhang et al., 2018). Since HMGB2 has a role in male fertility and spermatogenesis in other species (Ronfani et al., 2001; Li et al., 2022), we sought to determine its role in parasite sexual stage biology and transmission to the mosquito vector.

## Material and methods

### Reagents

Unless stated otherwise, the molecular biology reagents were purchased either from Millipore Sigma, USA or Thermo Scientific, USA. All oligonucleotides were purchased from Integrated DNA Technologies, USA.

### *P. falciparum* culture and transfection

*P. falciparum* NF54 and *Pfhmgb2¯* parasites were cultured in accordance with standard procedures at 37°C and supplemented with “malaria” gas containing 5% O_2_/5% CO_2_/90% N_2_. Asexual cultures were set up at 5% hematocrit while gametocyte cultures were set up at 4% hematocrit using O^+^ human red blood cells (Valley Biomedical, VA, US) and fresh medium was repleted daily. All cultures were maintained with complete RPMI media supplemented with either 0.5% AlbuMAX™ II (Thermo Scientific) medium as asexuals or 10% (v/v) type O^+^ human serum (Valley Biomedical, VA, US or Interstate Blood Bank, TN, US) as gametocytes. Gametocyte cultures were set up at 1% ring stage parasitemia and were maintained in six well plates with a final volume of 5 mL using methods published elsewhere (Tripathi et al., 2020).

**Figure 1:**
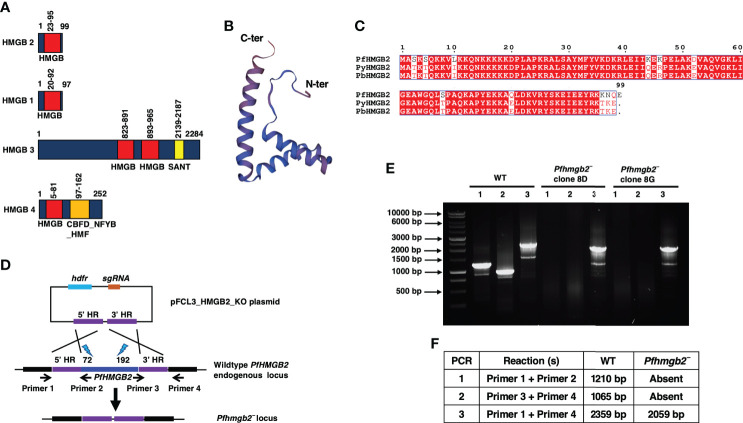
*Pf*HMGB2 structural features and generation of *Pfhmgb2¯*parasites *via* CRISPR/Cas9. **(A)** Schematic for the *Pf*HMGB2, *Pf*HMGB1, *Pf*HMGB3 and *Pf*HMGB4 proteins. *Pf*HMGB2, *Pf*HMGB1 and *Pf*HMGB4 have a single HMG box (red bar) domain while *Pf*HMGB3 has two. *Pf*HMGB3 and *Pf*HMGB4 also contain additional domains; SANT domain in *Pf*HMGB3 (yellow bar) and CBFD_NFYB:HMF domain in *Pf*HMGB4. **(B)** Predicted three-dimensional structure of *Pf*HMGB2. PDB template used 2mrc.1.A. The N and C terminus are indicated. **(C)** Sequence alignment for *Pf*HMGB2, *Py*HMGB2 and *Pb*HMGB2. Conserved residues are in white font on a red background. *Pf*-*Plasmodium falciparum*, *Py*-*Plasmodium yoelii*, *Pb*-*Plasmodium berghei*. **(D)** The schematic shows the strategy for disrupting the *PfHMGB2* gene. pFCL3_HMGB2_KO plasmids has homology regions from 5’ (5’HR) and 3’ (3’HR) of *PfHMGB2* locus, single guide RNA sequence (sgRNA) and human dihydrofolate reductase (hDHFR) locus cloned. Lightning bolts and numbers next to them indicate target site of gRNAs. **(E)** Diagnostic PCR for the confirmation of *PfHMGB2* deletion. The oligonucleotides were designed from outside 5’HR and 3’HR and *PfHMGB2* locus and positions are indicated by arrows in **(D)**. **(F)** The expected sizes for different set of PCRs are indicated.

Oligonucleotides used in the generation and genotyping analysis of *P. falciparum Pfhmgb2¯* parasites are mentioned in **Table 1**. Utilizing CRISPR/Cas9 strategy, the *PfHMGB2* locus (PlasmoDB gene identifier PF3D7_0817900) was deleted by double crossover homologous recombination. The pFCL3_HMGB2_KO 1 plasmid was generated through the ligation of a 20-nucleotide guide RNA sequence along with the complementary regions of *PfHMGB2* flanking both ends of the open reading frame. Similarly, pFCL3_HMGB2_KO 2 was generated by cloning a different guide RNA sequence with same homology arms as pFCL3_HMGB2_KO 1. 100 µg each of these two plasmids were mixed and transfected into the NF54 ring stage parasites *via* electroporation at 310 V and 950 μF by using a Bio-Rad Gene Pulser II (Bio-Rad Laboratories, Hercules, CA). Transfected parasites were selected using 8 nM WR99210 (gifted by Jacobus Pharmaceuticals). *PfHMGB2* deletion on clones obtained using limiting dilution cloning was confirmed *via* genotyping PCR (**Figures 1D–F**). To further confirm the gene deletion and absence of transcript, we prepared cDNA from WT and *Pfhmgb2¯* parasites using QIAGEN kit following manufacturer’s instruction and performed PCRs using *HMGB2* ORF oligonucleotides and 18s rRNA control oligonucleotides. Absence of a band for *HMGB2* in *Pfhmgb2¯* confirmed gene deletion.

**Table 1:** Oligonucleotides used in the study.

Oligonucleotides used for generation of *Pfhmgb2¯* parasites	
Oligo	Forward (5’-3’)
*Pf*HMGB2 5’Homo For	T**GCGGCCGC**CATATACATTTGTGTAGTTATATGTGTTACTATATATATGTTTAAGTA
*Pf*HMGB2 5’Homo Rev	CCAACCCGGGTATAGGCGCGCCTGAACTGGTCATAATATTCTGAACAATGCATATA
*Pf*HMGB2 3’Homo For	AGGCGCGCCTATACCCGGGTTGGCATTCAACATATATATGAATAAATATATATGCGTG
*Pf*HMGB2 3’Homo Rev	TAA**GTCGAC**CGATATAACTTAATATTTATGTTTACACCTTAAATTATGTTTCA
*Pf*HMGB2 Guide 1 For	**TATT**GTAGGCAGACAAAGCTCTCTT
*Pf*HMGB2 Guide 1 Rev	**AAAC**AAGAGAGCTTTGTCTGCCTAC
*Pf*HMGB2 Guide 2 For	**TATT**AAATTGATAGGTGAAGCTTG
*Pf*HMGB2 Guide 2 Rev	**AAAC**CAAGCTTCACCTATCAATTT
*Pf*HMGB2 Geno5 For	TTAATGTTATAATTTTTTGTTTTTCTTATTTATTTAAAATTTAAACAATTTTCTATAAAG
*Pf*HMGB2 Geno5 Rev	GCAACATCTTTTGCTAATTCTGGT
*Pf*HMGB2 Geno3 For	CAAAGTACGATATTCAAAAGAAATAGAAGAATATAGAAAG
*Pf*HMGB2 Geno3 Rev	CACATGTCTATAAATATGTTACTATTTTTATATATCATAAAACATACCC
18s For	AACCTGGTTGATCCAGTAGTCATATG
18s Rev	CCAAAAATTGGCCTTGCATTGTTAT
*Pf*HMGB2 ORF For	ATGGCTTCAAAATCTCAAAAGAAAGTATTAAAAAAACAAAAC
*Pf*HMGB2 ORF Rev	TTATTCTTGATTTTTCTTTCTATATTCTTCTATTTCTTTT

Bold residues refer to additional nucleotides added to oligonucleotides for cloning and plasmid preparation.

### Sequence analysis

*Plasmodium* DNA and protein sequences were retrieved from PlasmoDB (http://plasmodb.org/plasmo/). Domain analysis was done using SMART tool (http://smart.embl-heidelberg.de/).

### Measurement of asexual blood stage growth and gametocyte development

WT *Pf*NF54 and *Pfhmgb2*¯ parasites were synchronized at ring stages and were set up at 1% starting parasitemia and were maintained in 6-well plates as described above for comparative analysis of asexual blood stage development as well as gametocyte development. Asexual parasitemia was scored per 1000 erythrocytes after 48 and 96 hrs through Giemsa-stained thin blood smear microscopy. Gametocytemia per 1000 erythrocytes was scored on day 15 of *in vitro* culture, likewise through Giemsa-stained thin blood smear microscopy.

### Exflagellation, standard membrane feeding assay, oocyst and salivary gland sporozoite measurements

For analyzing exflagellation, equal volume of gametocytes from WT *Pf*NF54 and *Pfhmgb2¯* were mixed separately with human type O^+^ serum and O^+^ RBCs (50:50) % (v/v) and incubated at room temperature for 15 min. Exflagellation was scored for WT *Pf*NF54 and *Pfhmgb2¯* parasites *via* light microscopy by counting exflagellation centers in 10 optical fields of view at 40× magnification.

For SMFA, infectious blood meal was prepared by mixing stage V gametocytes for WT *Pf*NF54 or *Pfhmgb2¯* with human serum and O^+^ RBCs mixture (50:50) % (v/v) to achieve a final gametocytemia of 0.5%. Mosquitoes were fed as described in elsewhere (Tripathi et al., 2020). Following blood feeding, unfed mosquitoes were removed, and the rest were maintained for up to 20 days at 27°C, 75% humidity, and provided with 8% dextrose solution in 4-Aminobenzoic acid (PABA) water inside an incubator. *Anopheles stephensi* mosquitoes were dissected day 7 post blood meal for midguts and oocysts were enumerated under bright field microscope at 10× magnification. The mosquitoes were dissected day 14 post blood meal for salivary glands and sporozoites were enumerated using Neubauer’s chamber under bright field microscope at 40× magnification.

### Statistical analysis

Data collected was expressed as an average ± SD. Using unpaired two-tailed Student’s t test or Nested one-way ANNOVA test with one-way ANOVA, the statistical differences in data were determined. GraphPad Prism 9 was used to calculate significances, with values of p < 0.05 being considered as statistically significant. Significance is represented in the figures as either ns- not significant, p > 0.05; *p < 0.05; **p < 0.01; or ***p < 0.001).

## Results

### Generation of *Pfhmgb2¯* parasites

**Figure 2:**
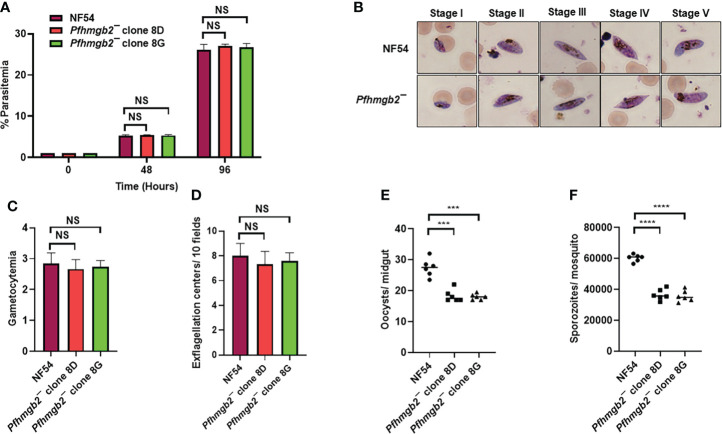
*Pfhmgb2¯* parasites grow normally as asexuals, undergo gametocytogenesis and show reduced transmission to the mosquito. **(A)** Ring stage synchronous cultures for WT *Pf*NF54 and *Pfhmgb2¯* (clone 8D and 8G) were set up at 1% parasitemia and parasite growth was measured over the course of two erythrocytic cycles using Giemsa-stained smears. Data were averaged from three biological replicates and presented as the mean ± standard deviation (SD). **(B)** WT *Pf*NF54 and *Pfhmgb2¯* parasites were tested for their potential to form gametocytes. Light microscopy of Giemsa-stained smears showing development of WT *Pf*NF54 and *Pfhmgb2¯* gametocytes and the five (I-V) distinct morphological stages. 1,000×magnifcation. **(C)** Day 15 gametocytemia for WT *Pf*NF54 and *Pfhmgb2¯* (clone 8D and 8G) was measured using Giemsa-stained smears. Data were averaged from three biological replicates and presented as the mean ± standard deviation (SD). **(D)** Number of exflagellation centers per field at 15 min post-activation WT *Pf*NF54 and *Pfhmgb2¯* (clone 8D and 8G). Data were averaged from three biological replicates and presented as the mean ± standard deviation (SD). **(E)** Oocysts per mosquito midgut were enumerated on day 7 post feed for WT *Pf*NF54 and *Pfhmgb2¯* (clone 8D and 8G) fed mosquitoes. Data were averaged from three biological replicates with a minimum of 50 mosquito guts and presented as the median. n = 3; biological replicates, **P < 0.05, Unpaired t-test; NS-Not significant. **(F)** Salivary gland sporozoites per mosquito midgut were enumerated on day 7 post feed for WT *Pf*NF54 and *Pfhmgb2¯* (clone 8D and 8G) fed mosquitoes. Data were averaged from three biological replicates with a minimum of 50 salivary glands and presented as the median. n = 3; biological replicates, ***P < 0.001, ****P < 0.0001, Unpaired t-test; NS-Not significant.

The *Pf*HMGB2 sequence was retrieved from PlasmoDB (https://plasmodb.org/plasmo/app) with gene identifier PF3D7_0817900. HMG box proteins normally have two HMG boxes, but *Pf*HMGB2 is atypical 99 amino acid (aa) protein with a single HMG box (23-95 aa) (**Figure 1A**). A comparison of all four HMGB proteins from *P. falciparum* revealed the differences between their domains. Like *Pf*HMGB2, *Pf*HMGB1 and *Pf*HMGB4 have a single HMG box domain while *Pf*HMGB3 contains two (**Figure 1A**). *Pf*HMGB3 and *Pf*HMGB4 also contain additional domains; SANT domain in *Pf*HMGB3 and CBFD_NFYB:HMF domain in *Pf*HMGB4 (**Figure 1A**). 3-dimentional structure prediction using SWISS-MODEL (https://swissmodel.expasy.org/interactive/G2dB9b/models/) showed that *Pf*HMGB2 has a conserved L-shaped structure like known eukaryotic HMG box proteins (**Figure 1B**). *Pf*HMGB2 shows very high similarity to rodent malaria parasite HMGB2 proteins (**Figure 1C**), indicating conservation of their function in the genus *Plasmodium*. Previous studies have indicated that *PfHMGB2* might be essential for asexual blood stages. This was assumed based on failure to obtain recombinant gene knockout parasites (Zhang et al., 2018; Lu et al., 2021). We however deleted the *PfHMGB2* gene using CRISPR/Cas9 mediated transgenesis (**Figure 1D**). Gene deletion parasites were confirmed by a set of diagnostic PCRs with oligonucleotide primers specific for the *PfHMGB2* locus and genomic regions 5′ (upstream) and 3′ (downstream) of the open reading frame (**Figures 1D–F**). Further PCRs were performed on cDNA prepared from WT *Pf*NF54 and *Pfhmgb2¯* parasites for HMGB2 ORF which confirmed deletion of the *PfHMGB2* locus (**Supplementary Figure 1**). To analyze the role of *PfHMGB2* in asexual blood stages, comparative growth assays were set up using two clones of *Pfhmgb2¯* (clone 8D and 8G) alongside wildtype (WT) *Pf*NF54 parasites. The growth was monitored over two replication cycles using Giemsa-stained thin smears prepared every 48-hr, which indicated that the growth rate of *Pfhmgb2¯* was similar to WT NF54 (**Figure 2A**). These results demonstrate that HMGB2 is dispensable for asexual parasite replication.

### *Pfhmgb2¯* parasites are transmissible to the mosquito vector and produce viable oocysts

We next analyzed the ability of *Pfhmgb2¯* parasites to generate gametocytes. For this, WT and *Pfhmgb2¯* (clone 8D and 8G) gametocytes were cultured *in vitro* as described elsewhere (Tripathi et al., 2020). Percent gametocytemia was scored for all the cultures on day 15 of *in vitro* culture using Giemsa-stained smears. This analysis revealed that *Pfhmgb2¯* parasites were able to undergo gametocytogenesis (**Figure 2B**) and mature to stage V gametocytes and had similar gametocytemia as the WT NF54 parasites (**Figure 2C**). We next analyzed the ability of *Pfhmgb2¯* parasites to form motile male microgametes in exflagellation assays. For this, day 15 gametocyte cultures for WT and *Pfhmgb2¯* parasites were activated by addition of O^+^ human serum and a temperature drop from 37°C to room temperature (RT). After *in vitro* activation of the gametocytes, wet mounts were prepared and the number of exflagellation centers were evaluated using light microscopy at 40× magnification in ten random fields of view. We observed a similar number of exflagellation centers for *Pfhmgb2¯* and WT *Pf*NF54 (**Figure 2D**), indicating that male gamete formation was normal in *Pfhmgb2¯*. Previous studies on rodent malaria parasite *P. yoelii* have shown that *Py*HMGB2 has a critical role in *Py* oocyst development in the mosquito vector (Gissot et al., 2008). Since *Pf*HMGB2 shows a very high degree of similarity and conservation with *Py*HMGB2 (**Figure 1B**) and not with HMGB3 or HMGB4, we aimed at investigating a potential effect of *PfHMGB2* deletion on parasite transmission to *A. stephensi* mosquitoes. Infectious blood meals were prepared using standard methods for WT *Pf*NF54 and *Pfhmgb2¯* stage V gametocytes and were fed to mosquitoes using standard membrane feeders. Mosquitoes were dissected on Day 7 post blood meal for scoring midgut oocysts numbers. This revealed that *Pfhmgb2¯* clones produced lesser average oocyst numbers in mosquitoes than WT *Pf*NF54 (**Figure 2E**). The median oocyst number in *A. stephensi* infected with the WT parasites was 28 while in *Pfhmgb2¯* it was 18 (**Figure 2E**). Quantitative analysis of salivary gland sporozoites fourteen days post feed showed reduced number of sporozoites in mosquitoes fed with *Pfhmgb2¯* (**Figure 2F**), likely caused by reduced numbers of oocysts.

Taken together, these results indicate that *Pf*HMGB2 is dispensable for gametocytogenesis and gametogenesis but has a modest role in parasite transmission to the mosquito vector or oocyst development.

## Discussion

In this study, we investigated the role of the *Pf*HMGB2 in asexual blood stage and sexual stage development. *Plasmodium* species harbor four HMGB proteins HMGB1-4 which are highly conserved among different parasite species (Briquet et al., 2006) and [PlasmoDB]. While all four proteins are expressed during asexual stages, *Pf*HMGB2 and *Pf*HMGB4 also show enhanced expression during gametocytogenesis (Lu et al., 2021), suggesting a role in sexual stage development.

HMGB proteins contain HMG box domain and are highly conserved throughout evolution. The HMG box domain is composed of ~80 aa folded in three α-helices arranged in an L shape (Weir et al., 1993; Baxevanis and Landsman, 1995). In higher eukaryotes, many proteins contain HMG boxes, the majority of which are transcription factors and contain a single HMG-box (Thomas and Travers, 2001). Other proteins may contain up to 6 HMG-box domains such as for example Ubf1 (Russell and Zomerdijk, 2005). The HMGB proteins typically contain two boxes, A and B, along with basic N- and C-terminal extensions and a C-terminal acidic tail (Thomas and Travers, 2001). The HMG boxes A and B show differences in their sequences but have a well conserved L-shaped structure, and show differences in their DNA binding and bending abilities (Yoshioka et al., 1999). HMGB proteins may or may not act as transcription factors and do not have a DNA sequence preference. They typically assist other transcription factors by bending the cognate DNA sequence and altering the positioning of nucleosomes and thus controlling the level of transcription (Agresti and Bianchi, 2003; Längst and Becker, 2004). HMGB protein also function in chromosomal maintenance, possibly by telomere maintenance (Schrumpfová et al., 2011; Polanská et al., 2012). The *HMGB1* gene deletion in mouse embryonic fibroblasts (MEFs) leads to chromosomal abnormalities, moderate shortening of telomere lengths, and lower telomerase activity compared to the WT MEFs, while *HMGB2* gene deletion (*hmgb2¯*) MEFs show elevated telomerase activity suggesting their opposite effects on telomerase activity (Polanská et al., 2012). HMGB2 also has a major role in male fertility and spermatogenesis (Ronfani et al., 2001; Li et al., 2022). Recent reports suggest a role for *Pf*HMGB1 in the integrity of centromere/telomere-based chromosome organization and thus regulation of gene expression (Lu et al., 2021). Other *Pf* proteins (*Pf*HMGB2-HMGB4) have not been functionally characterized so far.

Here we show that *Pf*HMGB2 shows a high degree of conservation with rodent malaria parasite orthologs. While *Pf*HMGB2 is expressed in asexual blood stages, its expression is elevated in stage V gametocytes (Briquet et al., 2006; Kumar et al., 2008). *Pf*HMGB2 contains an N-terminal extension followed by HMG box and a shorter c-terminal region and display a conserved L- shaped structure which is similar to HMG box proteins, suggesting its DNA binding properties. Previous studies have in fact demonstrated that both *Pf*HMGB1 and *Pf*HMGB2 possess DNA binding properties (Briquet et al., 2006). The differences in domain architecture between the four *P. falciparum* HMGB proteins suggest differences in their target sequences in the genome. *Pf*HMGB1 gene deletion parasites showed local chromatin alteration and dysregulated gene expression (Lu et al., 2021). For functional characterization of *Pf*HMGB2, we created gene deletion parasites using CRIPSR/Cas9 based gene editing. In contrast to a previous report (Lu et al., 2021), *PfHMGB2* could be deleted in our studies. Failure to obtain gene deletion parasites in previous study may be due to selection of the guide RNA which vary in their efficiency during CRISPR/Cas9 mediated transgenesis. This work revealed that, despite high expression of *Pf*HMGB2 in both asexual and sexual stages, it is not required for asexual blood stage replication and gametocyte development. We further demonstrated that *Pfhmgb2*¯ parasites undergo normal exflagellation, suggesting normal microgamete formation. Mosquito feed performed on WT *Pf*N54 and *Pfhmgb2*¯ gametocytes revealed that *Pfhmgb2*¯ could transmit to mosquito vector, although the oocyst numbers and salivary gland sporozoite numbers were reduced in comparison to WT parasites. The *Pfhmgb2*¯ phenotype resembles the reduced transmissibility of *Pyhmgb2*¯ parasites (Gissot et al., 2008), although the defect was more severe in latter. This also suggests the similarity in HMGB2 function across different species in *Plasmodium*. The lack of a very strong observable phenotype in *Pfhmgb2*¯ parasites is surprising, given very high expression of *Pf*HMGB2 in mature gametocytes. We hypothesize that there could be redundancy in function of HMGB proteins in *Plasmodium*. There is a possibility that *Pf*HMGB1 can complement *Pf*HMGB2 function as both are expressed in asexual stages while *Pf*HMGB4, which is highly expressed in sexual stages, can complement *Pf*HMGB2 during gametocyte stages and transmission to the mosquito. It is also possible that *Pf*HMGB2 along with *Pf*HMGB1 have role in immunomodulation which cannot be measured under standard laboratory conditions for human infective parasites. This hypothesis is supported by the studies demonstrating that recombinant *Pf*HMGB1 and *Pf*HMGB2 are potent inducers of pro-inflammatory cytokines such as TNFα from mouse peritoneal macrophages (Kumar et al., 2008).

The sexual phase of *P. falciparum* life cycle represents a critical bottleneck and is required for transmission of the parasite from human host to mosquito. In this study, we show that *Pf*HMGB2 is dispensable for the asexual growth of parasite but has a role in parasite mosquito infection. Its conservation across various *Plasmodium* spp. strongly suggests it does have a significant role most likely in chromatin organization and gene expression. These hypotheses could be investigated in future studies.

## Data availability statement

The original contributions presented in the study are included in the article/**Supplementary Material**. Further inquiries can be directed to the corresponding authors.

## Author contributions

Conceptualization: SK. Methodology: SK. Investigation: SK. Visualization: SK, SHIK. Resources: SHIK. Supervision: SHIK. Writing-original draft: SK. Writing-review & editing: SK, SHIK. All authors contributed to the article and approved the submitted version.

## Funding

This work is supported by seed funds to S.H.I.K. by Seattle Children’s *via* award number 24010119.

## Acknowledgments

The authors acknowledge continuous support and availability of the *Anopheles stephensi* mosquitoes provided by the Arthropod containment lab (ACL) I and II at CGIDR, Seattle Children’s.

## Conflict of interest

The authors declare that the research was conducted in the absence of any commercial or financial relationships that could be construed as a potential conflict of interest.

## Publisher’s note

All claims expressed in this article are solely those of the authors and do not necessarily represent those of their affiliated organizations, or those of the publisher, the editors and the reviewers. Any product that may be evaluated in this article, or claim that may be made by its manufacturer, is not guaranteed or endorsed by the publisher.

## Supplementary material

Supplementary Figure 1 Confirmation of HMGB2 deletion. The cDNA was prepared from WT and *Pfhmgb2¯* clone 8D parasites and PCRs were performed using the oligonucleotides designed from *HMGB2* open reading frame (ORF) and control 18s rRNA oligonucleotides. The band sizes for different set of PCRs are indicated.
