# Supplementary figures and images for: PfHMGB2 has a role in malaria parasite mosquito infection
Source: Front Cell Infect Microbiol. 2022 Nov 25;12:1003214. doi: 10.3389/fcimb.2022.1003214 (PMC9732239; doi:10.3389/fcimb.2022.1003214)

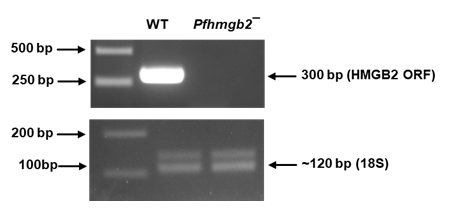

Supplement: Supplementary Figure 1 — Confirmation of HMGB2 deletion. The cDNA was prepared from WT and Pfhmgb2¯ clone 8D parasites and PCRs were performed using the oligonucleotides designed from HMGB2 open reading frame (ORF) and control 18s rRNA oligonucleotides. The band sizes for different set of PCRs are indicated. [file Image_1.tif]
