# Supplementary material for: PfHMGB2 has a role in malaria parasite mosquito infection
Source: Front Cell Infect Microbiol. 2022 Nov 25;12:1003214. doi: 10.3389/fcimb.2022.1003214 (PMC9732239; doi:10.3389/fcimb.2022.1003214)
Supplement: Supplementary file 2 [file Table_1.docx]

**Supplementary table 1.** Details of each mosquito infection experiment with WT NF54 and *Pfhmgb2¯* parasites.

| **Exp. number** | **Parasite** | **Number of mosquitoes** | **Prevalence (%)** | **Median Oocyst numbers** | **Sporozoite numbers per mosquito** |
| --- | --- | --- | --- | --- | --- |
| **1** | NF54 cage 1 | 50 | 94 | 25.5 | 56000 |
|  | NF54 cage 2 | 50 | 96 | 23.5 | 58800 |
|  | *Pfhmgb2¯* Clone 8D cage 1 | 50 | 98 | 17 | 36000 |
|  | *Pfhmgb2¯* Clone 8D cage 2 | 50 | 96 | 17 | 34800 |
|  | *Pfhmgb2¯* Clone 8G cage 1 | 50 | 88 | 18 | 31290 |
|  | *Pfhmgb2¯* Clone 8G cage 2 | 50 | 90 | 17 | 34400 |
| **2** | NF54 cage 1 | 50 | 98 | 28.5 | 61000 |
|  | NF54 cage 2 | 50 | 98 | 32 | 63200 |
|  | *Pfhmgb2¯* Clone 8D cage 1 | 50 | 94 | 22 | 41800 |
|  | *Pfhmgb2¯* Clone 8D cage 2 | 50 | 98 | 19 | 39500 |
|  | *Pfhmgb2¯* Clone 8G cage 1 | 50 | 96 | 18.5 | 33200 |
|  | *Pfhmgb2¯* Clone 8G cage 2 | 50 | 90 | 18 | 35300 |
| **3** | NF54 cage 1 | 50 | 100 | 27 | 61500 |
|  | NF54 cage 2 | 50 | 98 | 28 | 60900 |
|  | *Pfhmgb2¯* Clone 8D cage 1 | 50 | 98 | 18 | 35400 |
|  | *Pfhmgb2¯* Clone 8D cage 2 | 50 | 96 | 17 | 31900 |
|  | *Pfhmgb2¯* Clone 8G cage 1 | 50 | 92 | 17 | 38400 |
|  | *Pfhmgb2¯* Clone 8G cage 2 | 50 | 96 | 19.5 | 41300 |
